# Acute Effects of Soleus Stretching on Ankle Flexibility, Dynamic Balance and Speed Performances in Soccer Players

**DOI:** 10.3390/biology11030374

**Published:** 2022-02-26

**Authors:** Shi Huang, Hong-Jia Zhang, Xin Wang, Winson Chiu-Chun Lee, Wing-Kai Lam

**Affiliations:** 1 Sports Training Department, Shenyang Sport University, Shenyang 110102, China; shihwang@126.com; 2 College of Human Kinesiology, Shenyang Sport University, Shenyang 110102, China; 3 School of Mechanical, Materials, Mechatronic and Biomedical Engineering, University of Wollongong, Wollongong, NSW 2522, Australia; ccwlee@uow.edu.au; 4 Sports Information and External Affairs Centre, Hong Kong Sports Institute, Sha Tin, Hong Kong

**Keywords:** stretching, football, agility, dynamic stretch, leg strength, dynamic balance, curved running

## Abstract

**Simple Summary:**

Calf muscles, including the gastrocnemius and soleus muscles, are very important for soccer players, but the benefits of soleus stretching are rarely reported. Both regular and soleus stretching conditions lead to better ankle range of motion, maximum plantarflexion strength, dynamic balance and speed performances compared to the no-stretching control. Adding soleus stretches into the regular stretching protocol would allow further improvement of ankle flexibility, maximum plantarflexion strength and curved running speed performances. The results from this study can provide insights for pre-exercise design and subsequent performances in soccer.

**Abstract:**

Most dynamic stretching protocols include the gastrocnemius muscle, but soleus stretches are often neglected, which is the key powerful muscle for the push-off (concentric) of all speed movements. The purpose of this study was to examine whether the added soleus stretch in a regular stretching protocol would have greater benefits for ankle flexibility, dynamic balance and functional performance. Fourteen healthy male soccer players received each of the stretching conditions (regular stretching only (Regular), regular stretching with soleus stretching (Soleus) and no stretching (Control)) randomly on different training days, with two-day separation. The ankle flexibility, standing heel-lift balance and speed performances were assessed following each stretching intervention. The active dynamic stretches were performed for 30 s with three repetitions on each of the three (Regular) and four (Soleus) muscles. One-way ANOVA with repeated measures (or the Friedman non-parametric test) was performed to determine any significant effect with alpha = 0.05. Our findings revealed that both the Regular and Soleus stretching groups showed an increased active range of ankle motion compared to the no-stretching control (*Ps* < 0.05). In the heel raise balance test, both stretching groups experienced a significant increase in maximum plantarflexion strength as well as resultant anterior–posterior and medial–lateral CoP excursions compared to the no-stretching control (*Ps* < 0.05). In the sprinting tasks, both the Soleus and Regular stretching groups induced faster linear and curved running times (*Ps* < 0.05). When comparing the two stretching groups, Soleus stretching led to better ankle flexibility, maximum plantarflexion strength and curved running time (*Ps* < 0.05). Thus, added stretches on the soleus muscles can provide further benefits to speed performances in soccer.

## 1. Introduction

Soccer is a sport that requires lower limb flexibility to provide speed, coordination and agility [1,2]. Joint flexibility refers to the range of motion (RoM) available in a joint to move effectively, without inducing injury [3,4]. It was reported that soccer players with s lower pre-season hip and knee RoM are susceptible to a statistically higher risk for muscle strain injury to these muscle groups during a competitive season compared to players with a larger RoM [5]. Static and dynamic stretching exercises are typically recommended to players of amateur and professional levels before performing any sport activities.

Scientific evidence has shown that acute stretching increases the RoM and the length of musculotendinous units and improves mechanoreceptor reflexes [6,7], although no consensus was reached regarding motor task performances [6,8,9,10,11]. The inconsistent findings could be related to the use of different frequencies/durations [12,13,14] and types of stretching [6,7]. In addition, studies have shown that the positive effect of stretching is related to the increase in human core temperature [15].

Dynamic stretching of lower limb muscles such as the gluteus, hamstrings, quadriceps and calf muscles increases joint flexibility, thereby producing a positive acute effect on sprinting [15,16], vertical jumping [16,17], agility performance [16,18], ball handling and kicking accuracy compared to no stretching and static stretching [19,20]. Most previous studies investigated the effects of stretching multiple muscle groups [16,18,20,21] and the muscles around the hip and knee joints [18,22]. Very few investigations have been established on calf muscles in soccer players, and many studies on calf stretching have focused only on the gastrocnemius muscle while neglecting the soleus muscle [16,18,20,23,24]. Ankle flexibility is associated with dynamic balance ability [1,25,26], which is a key contributing factor for ball control, kicking accuracy and speed and agility performances [19,20,27,28]. Therefore, stretching the calf muscles would greatly benefit soccer performances.

The soleus is a single-joint muscle which produces action (plantarflexion) at the ankle joint solely. This is a powerful muscle that works with the gastrocnemius to accomplish all types of weight-bearing activities, such as speed and jump movements [29]. Many studies have focused on the stretching effect of the gastrocnemius muscle in the past, but the soleus muscle is less studied [15,17,18,20,21,26,30,31]. Knee flexion is necessary to create sufficient stretch of the single-joint soleus muscle [26]. Hence, the purpose of this study was to investigate the effect of added dynamic soleus stretching on ankle flexibility, dynamic balance, calf strength and speed performances. It was expected that the dynamic stretching exercises (regular and additional soleus) would improve those performances and that the added soleus stretching condition would induce the greatest improvements. This information can help to identify better stretching protocols for the lower limbs, which is insightful for coaches and sports scientists who are considering improving ankle flexibility in training and game plays.

## 2. Materials and Methods

### 2.1. Participants

Based on the previously reported large effect sizes between stretching types [32], we calculated a priori power in G-Power software with an alpha of 0.05 and a power of 0.8 and estimated that 14 participants would be adequate for this study. This is similar to other performance studies that compared types of stretching (e.g., *n* = 12 males [33]; *n* = 10 males [34]; *n* = 14 females [32]). Fourteen male university soccer players (mean (SD) age: 22.6 (1.7) y; height: 174.9 (5.8) cm; weight: 72.2 (11.6) kg) participated in this study. All participants were right leg dominant and had no lower extremity injuries in the past six months. They did not perform any strenuous exercises within 48 h prior to the study. They had more than four years of soccer training experience at the national second-tier level. All participants received soccer training for more than 5 h per week. Written informed consent was obtained from each participant, and ethical approval was provided by the institutional Human Research Ethics Committee.

### 2.2. Stretching Conditions

**Figure 1:**
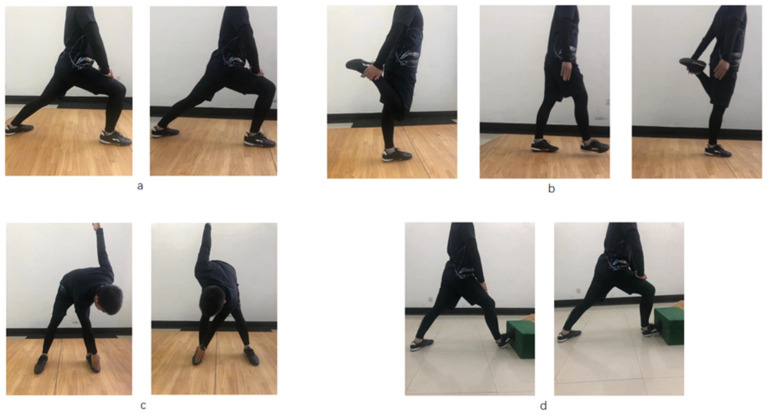
Stretching exercises with four muscles: (**a**) gastrocnemius; (**b**) quadriceps; (**c**) hamstring; (**d**) soleus. The regular stretching condition involved muscles (**a**–**c**) and the soleus stretching condition involved muscles (**a**–**d**).

There were three stretching conditions in this study, namely regular stretching only (Regular), regular stretching with added soleus stretching (Soleus) and a control (no-stretching condition). The two stretching protocols involved dynamic stretching, the descriptions of which are provided in Table 1 and Figure 1. For the regular stretching condition, participants were instructed to perform three repetitions for about 30 s of dynamic stretches of the hamstring, quadriceps femoris and gastrocnemius muscles of each leg. They were given 1 min rest after the stretching of all muscle groups, in accordance with previous studies [21,35,36]. For the Soleus condition, participants were asked to perform regular stretching with additional stretching of the soleus muscle, with 30 s stretching for each of the four muscles (hamstring, quadriceps femoris, gastrocnemius muscle and soleus) [36]. For the control (no stretching) condition, the participants were instructed to sit for 8 min.

### 2.3. Evaluation Tasks

#### 2.3.1. Ankle Flexibility Test

**Figure 2:**
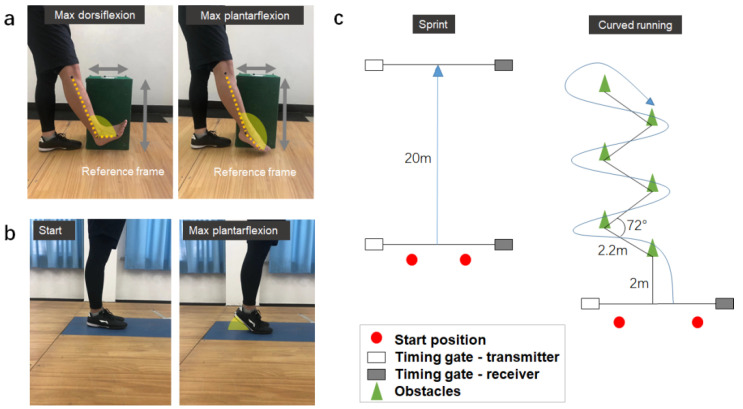
Experimental protocols for (**a**) flexibility test, (**b**) standing heel raise test (dynamic balance and muscle strength) and (**c**) functional performance test (sprinting and curved running).

Three reflective markers were placed over the fibular head, lateral malleolus and the base of the fifth metatarsal bone of the dominant side for video analysis [37]. Whilst standing straight on their left leg, the participants were required to fully extend their right knee and actively perform maximum ankle plantarflexion and dorsiflexion with three consecutive trials [38]. To capture the sagittal ankle motion, a mobile phone (iPhone X, Apple, USA, sampling at 60 Hz) was set perpendicular to the right leg at a distance of 1.5 m (Figure 2a), as described in previous studies [39,40]. The video analysis with the mobile phone application was proven to be reliable for measuring joint angle as it had a root mean square value under 0.3 degrees when compared with the actual industrial robotic data [40]. To accurately calculate the ankle motion, a reference frame with known height and width was used for calibration. The footage was analyzed in SIMI (Simi v8.5.6, Simi, Germany) to calculate the joint angle between the lower leg and foot segments. The active RoM (maximum dorsiflexion minus maximum plantarflexion) was selected for further analysis [5,37,38]. The average RoM value was calculated from the three successful trials for further comparisons between stretching conditions.

#### 2.3.2. Standing Heel Raise Test (Dynamic Balance and Muscle Strength)

The heel raise test used in this study (Figure 2b) is commonly used to evaluate both dynamic balance and muscle strength [41]. The participants were asked to stand on a force plate (AMTI BP4002000, Watertown, MA, USA, sampling at 1000 Hz) to perform the heel raise task with both legs and feet in contact with each other [41,42]. The participants were required to perform a heel lift as quickly as possible without loss of balance and then put their feet down naturally. We measured the excursion of the center of pressure (CoP) during the ascending/push-off phase (i.e., plantarflexion) of the heel raise task to evaluate the dynamic balance of the human body. The posturography data, including maximum anterior–posterior (AP), medial–lateral (ML) and resultant CoP excursions, were determined to evaluate dynamic balance during the heel raise task [43,44,45]. In brief, we first defined the origin as the most posterior point of the CoP trajectory. Then, AP and ML deviations were determined with respect to the same (*y*-axis) and perpendicular (*x*-axis) to the longitudinal foot axis, respectively. The y-coordinates (AP locations of CoP) and x-coordinates (ML locations of CoP) were extracted to determine the largest absolute differences in respective AP and ML directions throughout the upward heel lift phase. Furthermore, the resultant CoP excursion was calculated by summing the absolute differences between the x-coordinates and CoP path and between the y-coordinates and CoP path. The peak vertical GRF was determined to indicate the maximum plantarflexion force (i.e., lower leg strength) during the heel raise task [43,46]. The CoP excursion and GRF data were normalized by body height and body mass, respectively. Three successful trials were conducted to determine the CoP and maximum plantarflexion force parameters for subsequent analyses.

#### 2.3.3. Functional Performance Test

Sprinting and curved running tasks were used to assess the functional performances for this study (Figure 2c). Both of the tasks were carried out on a soccer field (100 × 55 m). Two pairs of timing gates (Speed Tech, S-001, China) were set at the start and end positions for each task (Figure 2c). The participants were asked to initiate the movement at the starting line and complete the tasks at their maximum effort [16]. All participants wore the same soccer clothing and shoes (Li Ning, Beijing, China) throughout the functional tests. Three successful trials were conducted for each of the two functional performance tasks, and the best trial (i.e., the fastest completion trial) was selected for subsequent analysis. A 2-min break and 3-min break were provided between trials and between tasks, respectively.

### 2.4. Procedure

**Figure 3:**
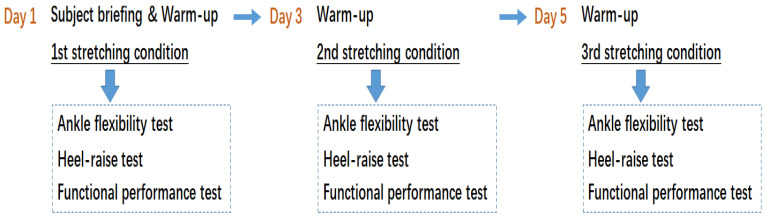
Experimental procedure over three test days. The regular stretching, soleus stretching and control conditions were randomly assigned across participants.

All participants were randomized for intervention order prior to the three testing sessions to limit the confounding effect of test learning on outcome. The order of the stretching conditions was randomized. Participants drew lots to determine the sequence of stretching and testing tasks. All participants attended three sessions (Day 1, Day 3 and Day 5) with 48 h separation time (Figure 3) [21], performing each of the three stretching conditions (regular stretching, soleus stretching and control) on each day. The participants warmed up by jogging two laps of a standard soccer field, which lasted about 5 min before each session. Then, participants were asked to perform the stretch, followed by the ankle flexibility, heel raise and functional performance tests. Prior to the stretching, written instructions and visual demonstrations were provided for each of the participants (Table 1 and Figure 1). The participants were asked to perform active stretches for 30 s on each of the muscles until they reached their maximum range of motion for each joint. For the control condition, the participants were asked to sit and wait for the evaluation tests, without performing any stretching on the lower limb muscles.

Before the evaluation tests, 5 min rest was provided after the stretching session. For the ankle flexibility test, the participants were asked to perform maximum dorsiflexion and plantarflexion. For the heel raise test, the participants were asked to perform a heel lift as quickly as possible without loss of balance. For the functional performance tests, the participants were instructed to complete the tasks at their maximum effort. To minimize physical fatigue, three trials were conducted for each test [47]. All movement and evaluation test conditions were randomly assigned across participants.

### 2.5. Data Analysis

All statistical analyses were performed with the SPSS 20.0 program. The normality and homogeneity of variance were assessed by P-P diagram and the Shapiro–Wilk test and Levene’s homogeneity test, respectively. If a variable was regarded as normal and having homogeneity of variance, one-way ANOVA with repeated measures was performed to test any stretching effect. Otherwise, the Friedman non-parametric test was performed followed by the Wilcoxon signed-rank test. Pairwise comparison was then performed when the *P*-value was less than 0.05. The effect size was determined according to the *r*-value, and the level of effect size was regarded as small (*r* < 0.3), medium (0.3 < *r* < 0.5) and large (*r* > 0.5) [43].

## 3. Results

The statistical results indicate significant effects of stretching on all of the tested variables (all *P-*values < 0.05, medium to large effect; Table 2). Significant increases in ankle RoM were found in the Soleus (*P* < 0.001) and Regular stretching groups (*P* = 0.008) compared with the no-stretching control. In the heel raise test, both the regular and soleus stretching conditions led to larger maximum plantarflexion forces (*Ps* < 0.001), resultant CoP (*Ps* < 0.001) and AP CoP (*Ps* < 0.05) and ML CoP excursions (*Ps* < 0.05) than the no-stretching control. In the functional performance tests, the soleus (*P* < 0.001) and regular stretching (*P* < 0.001) conditions induced faster sprinting times than the control, and the soleus condition induced faster curved running times (*P* < 0.001) than the control.

When comparing the differences between soleus and regular stretching, participants performing soleus stretching demonstrated larger ankle RoM (*P* = 0.008), larger maximum plantarflexion force (*P* < 0.001) and faster curved running time (*P* < 0.001) than the regular stretching group participants. There were no significant differences between the two stretching conditions in all CoP variables (*Ps* > 0.05).

## 4. Discussion

This study examined the effect of the added soleus stretch into regular stretching on ankle flexibility, plantarflexion strength, dynamic balance and speed performances. Our results revealed that significant improvements in ankle RoM, maximum plantarflexion force and soccer-specific performances were found in both stretching conditions (regular and soleus stretching) compared to the no-stretching control. Furthermore, when compared with the regular stretching condition, the addition of soleus stretches to the regular stretching protocol induced greater benefits to improve ankle joint range of motion, maximum plantar force and curve running time, which are considered important aspects of functional performance in soccer [1,19,20,25,26,27,28]. Since the soleus muscle works together with the gastrocnemius in all types of weight-bearing activities, implementing soleus stretches would help to reduce tightness and cramping of the lower legs and allow for additional improvement in ankle RoM, which is associated with better sport performances [1,2] and reduced injury [3,4,5].

Our results show that both the regular stretching and soleus stretching protocols (i.e., regular stretch with additional soleus stretches) improved the flexibility of the ankle joint, which generally agrees with previous studies on dynamic stretching of different muscle groups [48,49]. During pre-activity preparation, dynamic stretching was found to be beneficial as it increased muscle power production in soccer [15,16,19,23,30,50], basketball [17,51] and track-and-field [8] performances, while static stretching was consistently shown to have muscle power deficits [15,17,18,20,21,26,30,31,52]. The increase in the active RoM of the ankle in our study can be explained by the changes in physiological structure, toe flexion and dorsiflexion, which are the determinants contributing to the ankle RoM [38]. In our study, adding a 30-s soleus stretch to the regular stretching protocol further improved the ankle RoM compared to the regular stretch protocol, which stretched only the gastrocnemius muscle. Stretching the soleus muscles could be considered to improve ankle flexibility [36].

Improved joint RoM is believed to be associated with better dynamic balance, muscle force generation and sports performances [15,18,53,54]. Our findings indicated a larger maximum plantarflexion force and larger CoP excursion during the heel raise task following both the soleus stretching and regular stretching protocols, indicating the functional benefits from the dynamic stretching. This is generally supported by previous studies, which showed that changes in ankle RoM have the greatest influence on CoP excursion and dynamic balance [1,24,54,55]. This is also in line with previous studies which showed that dynamic stretching has a beneficial effect on lower limb muscle strength [22,33,56]. In a study conducted involving an explosive rebound task [22], the dynamic stretching protocol induced higher explosive lower limb power compared to the no-stretching control. Furthermore, dynamic stretching protocols, but not static stretching, enhance muscle performance [46]. When compared with static and PNF stretching, Franco et al. [46] found that dynamic stretching led to the greatest increment in maximum anaerobic strength. The current results from our dynamic stretch routines (i.e., regular or soleus stretch protocol) show a positive effect on maximum plantarflexion force, with larger forces generated from soleus stretching than from regular stretching. Consistent with previous studies [22,33,56], the results of the strength test show that dynamic stretching is beneficial to the improvement of lower limb strength, and stretching the soleus muscle will have a significant positive effect. A possible reason is that dynamic stretching will strengthen the active contraction ability of the muscle and increase its strength. Moreover, the elastic potential energy of the Achilles tendon will be released after dynamic stretching, leading to a better force generation effect. Extra stretching of the soleus will help this process by strengthening this muscle. This can provide some insight to develop a stretching protocol for acceleration- and speed-related performances.

The increased CoP in our dynamic heel raise task can be considered a sign of better dynamic balance [41,45]. Kim et al. [45] found that the CoM displacement and CoP excursion for increased reach distance in a Y-balance task were improved across five-day balance training, suggesting an improvement in dynamic balance. Chatzopoulos et al. [21] examined the dynamic balance of athletes in different stretching types using a stable platform and showed that dynamic stretching was more effective to enhance the dynamic balance. The increase in CoP could be explained by the increased range of plantarflexion (i.e., higher heel raise) or faster plantarflexion (i.e., larger plantarflexion force). There were no significant differences between the two stretching conditions in terms of all CoP variables, suggesting that the additional soleus stretch may not influence the CoP in the straight-knee heel raise task.

Regarding soccer-specific performances, our participants had faster sprinting and better curved running performances after performing soleus stretching, whereas the regular stretch condition group showed a better performance only in sprinting when compared to the no-stretch control. Our positive results of sprinting performance are similar to previous studies [16,23,24]. Little and Williams [23] compared the effects of static stretching, dynamic stretching and a no-stretching control on 20 m sprinting performance and found that professional footballers performing the dynamic stretching protocol demonstrated faster speeds than those in the other two conditions. For the curved running task, our results are also in line with the previous studies [3,16,21,23,24].

All of these studies concluded that dynamic stretching was effective for curved running performance. Interestingly, additional stretching of the soleus muscle had an added benefit over the regular dynamic stretching protocol for curved running but not the straight sprinting task. This could be explained by the difference in knee joint position during the push-off step between straight sprinting and curved running movements. Typically, the knee joint is in a fully extended position during push-off of a sprint, whereas it is more flexed during push-off of a curved running step. The gastrocnemius is a two-joint muscle and could be more dominant than the soleus to generate push-off power (i.e., powerful plantarflexion) during the straight-knee condition (straight sprinting) when compared to a more flexed-knee condition (curved running). Therefore, the effect of additional soleus stretching would be task- or knee-position-specific. Based on the above research results, it is confirmed that stretching of the soleus should be incorporated into the regular lower leg stretching protocol as indicated by the improved ankle RoM, ankle plantarflexion strength and curved running performances.

Some limitations should be considered when interpreting our results. First, we assessed only 14 young healthy male soccer players. A larger number of participants could increase the statistical power to detect a small difference that is likely meaningful in maximum sport performances. The results may also not be generalizable to females and non-soccer players due to the potential differences in anthropometry and strength as well as ligamentous and muscle compositions. Second, only the short-term effect was investigated in this study. Longitudinal investigations should be considered before a viable conclusion can be made. Third, all the measurements were carried out in extended knee position. With the use of a joint goniometer and EMG, we would require participants to perform maximum plantarflexion with various knee flexion angles in order to differentiate the force contributions from the soleus and gastrocnemius muscles. Finally, as very few studies have investigated the effect of soleus stretching, direct comparison with our results is not possible. In the future, studying the dynamic soleus stretching duration and frequency as well as the stretching effect on various types of movements (e.g., jumping, long-distance run) should be considered to provide insightful information for designing stretching protocols for different sports.

## 5. Conclusions

Both dynamic stretching protocols (soleus and regular conditions) are effective in improving ankle flexibility, maximum plantar force and soccer-specific performance compared to the no-stretching control. Participants performing additional soleus stretching showed a larger ankle RoM, leading to greater plantarflexion strength and better curved running performance compared with those performing the regular stretching protocol. Based on the results from this study, incorporating soleus stretching into the regular stretching protocol is recommended for improved plantarflexion strength and athletic performance.

## Figures and Tables

**Table 1:** Stretching description.

Muscle Group	Stretching Description
Gastrocnemius	Stand and take a step forward while keeping both hands on knee of the front foot and the upper body upright. Then, move the front knee forward and keep the heel of the back foot (stretching leg) on the ground.
Hamstrings	Stand on the floor with both legs together. Then, bend the trunk forward to touch each foot with hands.
Quadriceps	Stand still with the supporting leg. Then, grasp the raised foot (stretching leg) with one hand before pulling the heel towards the buttocks.
Soleus	Stand and take a step forward while keeping both hands on knee of the front foot and the upper body upright. Then, bend the knee of the front leg (stretch of the soleus) while standing on the back leg. Then, move the front knee forward (stretching leg) and keep the heel of the back foot on the ground.

**Table 2:** The statistical results for ankle flexibility, standing heel raise and speed performance tests under three stretching conditions (Raw GRF data can be found in Table S1).

	Control	Regular Stretching	Soleus Stretching	*p*	Effect Size (*R*)	Power (*Β/*Chi-Square)
**Flexibility test**						
^ Ankle RoM (deg)	58.6 (6.28)	63.0 (8.67) #*	67.8 (8.40) *	0.001	-	28.0
**Heel raise test**						
Maximum plantarflexion force (BW)	1.59 (0.28)	1.84 (0.34) #*	2.05 (0.33) *	<0.001	0.877	1.00
Resultant CoP excursion (BH)	24.2 (11.3)	40.3 (15.0) *	47.1 (19.3) *	<0.001	0.784	1.00
^ Maximum anterior–posterior CoP excursion (BH)	20.0 (10.1)	31.5 (16.6) *	34.3 (17.6) *	0.008	-	9.57
^ Maximum medial–lateral CoP excursion (BH)	5.3 (3.5)	6.8 (3.1) #*	9.2 (5.2) *	0.003	-	11.8
**Functional performance test**						
20-m sprint (s)	3.23 (0.16)	3.13 (0.18) *	3.12 (0.16) *	0.005	0.584	1.00
Curved running (s)	12.78 (0.63)	12.58 (0.72) #	12.24 (0.52) *	0.001	0.467	1.00

^ indicates Friedman test performed. * indicates significant difference from control (*p* < 0.05). # indicates significant difference between Soleus and Regular stretching conditions (*p* < 0.05). RoM = range of motion; CoP = center of pressure.

## Data Availability

The GRF data are provided as a supplementary document for this study.

## Supplementary Materials

The following supporting information can be downloaded at: https://www.mdpi.com/article/10.3390/biology11030374/s1, Table S1: The Raw and processed GRF data.
